# Behavioral disturbances in porencephaly. Report of a case

**DOI:** 10.1192/j.eurpsy.2021.1948

**Published:** 2021-08-13

**Authors:** L. Soldado Rodriguez, M. ValverDe Barea, M.O. Solis

**Affiliations:** 1 Mental Health Unit, Complejo Hospitalario de Jaen, Jaen, Spain; 2 Jaén, Complejo Hospitalario Jaén, Jaén, Spain

**Keywords:** Porencephaly, emergency, confusional syndrome, Neuroscience

## Abstract

**Introduction:**

Porencephaly is a neurological condition that can develop before or after birth, characterized by cysts located in any place inside the brain parenchyma, which generally are covered by plain walls and encircled by an atrophic crust. It generates a very variable clinic appearance, with severe cases of high disability and slight cases with a light neurological involvement, which also can go unnoticed until adulthood. The prevalence is unknow and the inheritance is autosomal dominant Male patient of 45 years diagnosed with porencephaly with cerebral palsy that affects left half and cognitive disability. His father reports an emerging defiant behavior, mutism and decrease of appetite from a week ago. No triggering stress factors are reported.

**Objectives:**

Show the importance of include in the differential diagnose hypoactive confusional syndrome.

**Methods:**

On urgent medical visit, male comes with ataxic gates which wasn’t shown before. Inhibited attitude, semiflexed staring at floor, with sparing and monosyllabic speech answers, verbalizing discomfort and personal concern. Sleep-wake rhythm disruptions.

**Results:**

Blood tests and drug screening shows no abnormalities Cranial CT: Without acute lesion Urinary infection observed.

**Conclusions:**

It is important to make complementary test to exclude organic frames which could justify acute-subacute psychopathology. In this case, diagnosis was acute confusional syndrome, however, most known presentation is the hyperactive one which include motor hyperactivity, inappropriate behavior or disorganization and alterations of sensory perception. Hypoactive must always be considered, which is the concluding diagnosis in this case.

**Disclosure:**

No significant relationships.

